# Modulation of the extrinsic cell death signaling pathway by viral Flip induces acute-death mediated liver failure

**DOI:** 10.1038/s41419-019-2115-y

**Published:** 2019-11-21

**Authors:** Miriam Bittel, Andreas E. Kremer, Michael Stürzl, Stefan Wirtz, Iris Stolzer, Markus F. Neurath, Gianna Ballon, Claudia Günther

**Affiliations:** 1 Department of Medicine 1, University hospital, Friedrich-Alexander-University Erlangen-Nürnberg, Erlangen, Germany; 2 Department of Surgery, Division of Molecular and Experimental Surgery, University hospital, Friedrich-Alexander-University Erlangen-Nürnberg, Erlangen, Germany; 3 Department of Pathology and Laboratory Services, Cooper University Health Care, Camden, NJ USA

**Keywords:** Apoptosis, Necroptosis, Liver diseases, Experimental models of disease

## Abstract

During viral infections viruses express molecules that interfere with the host-cell death machinery and thus inhibit cell death responses. For example the viral FLIP (vFLIP) encoded by Kaposi’s sarcoma-associated herpesvirus interacts and inhibits the central cell death effector, Caspase-8. In order to analyze the impact of anti-apoptotic viral proteins, like vFlip, on liver physiology in vivo, mice expressing *vFlip* constitutively in hepatocytes (*vFlip*^*AlbCre+*^) were generated. Transgenic expression of *vFlip* caused severe liver tissue injury accompanied by massive hepatocellular necrosis and inflammation that finally culminated in early postnatal death of mice. On a molecular level, hepatocellular death was mediated by RIPK1-MLKL necroptosis driven by an autocrine TNF production*.* The loss of hepatocytes was accompanied by impaired bile acid production and disruption of the bile duct structure with impact on the liver-gut axis. Notably, embryonic development and tissue homeostasis were unaffected by *vFlip* expression. In summary our data uncovered that transgenic expression of *vFlip* can cause severe liver injury in mice, culminating in multiple organ insufficiency and death. These results demonstrate that viral cell death regulatory molecules exhibit different facets of activities beyond the inhibition of cell death that may merit more sophisticated in vitro and in vivo analysis.

## Introduction

Viruses are involved in many pathogeneses of the human liver^1–3^. In order to defend the body from the invading pathogen, infected liver cells undergo programmed cell death to eliminate the pathogen followed by an recruitment of immune cells, resulting in acute liver damage and inflammation^4–6^. An persistent virus-mediated inflammatory cell death response can result in chronic inflammation and potentially progression to hepatic fibrosis, cirrhosis, and oncogenesis^2,3,7^.

Therefore, a more profound understanding on the molecular mechanisms of programmed cell death in the context of liver diseases is crucial in order to identify prospective therapeutic targets^8,15^. In particular, since the classical categorization in regulated apoptosis- and unregulated necrosis-mediated cell death has recently been challenged by the discovery of regulated necrosis^8^. Regulated necrosis exhibits the characteristic morphological hallmarks of necrosis, but describes several different forms of programmed cell death with distinct signaling pathways, such as necroptosis, pyroptosis, ferroptosis, and NETosis^8,9^.

Caspase-8 has an important function as key switch between apoptosis and necroptosis, as well as clearance of infected cells^10–15^. Therefore, Caspase-8 is a strong target for viral cell death regulatory proteins^16^. One of these cell death regulators expressed by several herpes and poxviruses is viral FLIP (vFLIP)^17,18^, a Caspase-8 inhibitor and NF-κB activator^19^.

Here we provide functional evidence that transgenic expression of vFLIP in hepatocytes is sufficient to promote excessive hepatocellular death mediated by regulated necrosis.

## Material and methods

### Animals and Housing

The generation of mice carrying a FLAG-tagged HHV8-vFlip flanked by a loxP-flanked neo^R^-STOP cassette and a frt-flanked IRES-GFP sequence in an ubiquitously expressed ROSA26 locus (Rosa26.vFlip mice) was described earlier^20^. Transgenic mice expressing vFlip in hepatocytes were generated by breeding Rosa26.vFlip mice to Albumin-Cre (AlbCre) mice (B6.Cg-Speer6-ps1Tg(AlbCre)21Mgn/J) obtained from the Jackson Laboratory. Throughout the whole manuscript, we used AlbCre negative littermate controls to exclude strain-dependent differences in susceptibility. Reporter mice expressing tdTomato in hepatocytes were generated by crossing Rosa26.tdTomato, as described before^21^, to Albumin-Cre mice. Mice were routinely screened for pathogens according to FELASA guidelines.

### Histology and immunohistochemistry

Histopathological analyses were performed on formalin-fixed paraffin-embedded tissue after Mayer’s haematoxylin and eosin (H&E) staining. Immunofluorescence staining of tissue sections was performed using the Alexa Fluor and TSA Cy3/Fluorescein system as recommended by the manufacturer (Perkin&Elmer). The following antibodies were used: murine MLKL (Biorbyt, Cat.No.: orb32399), GSDMD (abcam, Cat.No.: ab209845), Cytokeratin 19 (abcam, Cat.No.: ab52625), cleaved CASP3 (Cell Signaling, Cat.No.: 9661S), Albumin (abcam, Cat.No.: ab106582), CD11c (BD Bioscience, Cat.No.: 550283), F4/80 (eBioscience, Cat.No.: 14-4801-85), Myeloperoxidase (MPO) (abcam, Cat.No.: ab9535), and secondary anti-rabbit (Dianova, Cat.No.: 111-065-144), anti-chicken (abcam, Cat.No.: ab150169), anti-Armenian hamster (BioLegend, Cat.No.: 405501), anti-rat (Bioscience, Cat.No.: 554014), or anti-rabbit-Alexa 647 (BioLegend, Cat.No.: 406414). Nuclei were counterstained with Hoechst 33342 (Invitrogen). Cell death (TUNEL) was analysed using the in-situ cell death detection kit (Roche). Images were obtained using a confocal fluorescence microscope (LEICA TCS SP5 II) or the microscope LEICA DMI 4000B together with the LEICA DFC360 FX or LEICA DFC420C camera and the imaging software “LAS AF” (Leica). Oil red staining was performed on cryosections as recommended by protocol described before^22^.

### Liver organoid culture system

Liver organoids were generated by digesting embryonic livers (E20) from *vFlip*^*AlbCre+*^ and control mice and liver progenitor cells were cultured in a HepatiCult™ Organoid Growth Medium (Mouse) (STEMCELL technologies) for 7 days. Premature liver organoids were treated with selected factors Necrostatin-1 (30 µM, Sigma-Aldrich), Etanercept (25 µg/ml Enbrel®, Pfizer), Interferon β + γ (100 ng/ml each, PeproTech) in addition to A83-01 (50 nM, Sigma-Aldrich) for hepatocyte maturation.

### Gene expression

Total RNA was extracted from hepatic-, intestinal tissue or liver organoids using the peqGOLD Total RNA/ MicroSpin Kit (Peqlab, Erlangen, Germany). cDNA was synthesized using the SCRIPT cDNA Synthesis Kit from Jena Bioscience (Jena, Germany) and analysed by SYBR Green-based real-time RT-PCR using gene-specific primers. PCR product specificity was verified by performance of a melting curve for each primer set. Experimental values were normalized to levels of the housekeeping gene hypoxanthine guanine phosphoribosyl transferase (*Hprt*). Primer sequences are available on request.

### Immunoblotting

Proteins were isolated from liver biopsies using Cell lysis buffer (Cell Signaling, Cat.No.: 9803) supplemented with 1 mM PMSF (Cell Signaling, Cat.No.: 8553). Lysates were centrifuged at 14,000 rpm for 20 min (4 °C). Proteins were separated using a MiniProtean-TGX gel (4–15% polyacrylamide; Bio-Rad) and transferred to a Nitrocellulose membrane (Bio-Rad). Membranes were probed with the following primary antibodies: GFP (abcam, Cat.No.: ab290), MLKL (Biorbyt, Cat.No.: orb32399), GSDMD (abcam, Cat.No.: ab209845), GPX4 (abcam, Cat.No.: ab125066), GAPDH-HRP (abcam, Cat.No.: ab9482) and HRP-linked anti-rabbit (Cat.No.: 7074) (Cell Signaling) was used as a secondary antibody.

### Clinical chemistry

Serum concentrations of aspartate aminotransferase (AST) and alkaline phosphatase (AP) were measured in the clinical chemistry unit of the University Medical Center Erlangen.

### Statistical analysis

Comparisons of two groups were performed using an unpaired two-tailed *t*-test. Comparisons among multiple groups were performed using ANOVA as outlined in the particular experiment with Tukey’s test as post hoc test. Data sets are displayed as Tukey boxplots (box from 25th to 75th percentile, line represents median; whiskers according to Tukey’s method) unless indicated otherwise and statistical significance was accepted with *p* < 0.05 (NS *p* ≥ 0.05; *p < 0.05; **p < 0.01; ***p < 0.001; ****p < 0.0001). All *p-*values calculated using Tukey’s test are given as multiplicity adjusted *p*-values. Statistical calculations were performed using GraphPad Prism 7 (GraphPad Software).

### Statement

All authors had access to the study data and reviewed and approved the final manuscript.

## Results

### Transgenic expression of *vFlip* induces acute liver failure and neonatal death

**Fig. 1:**
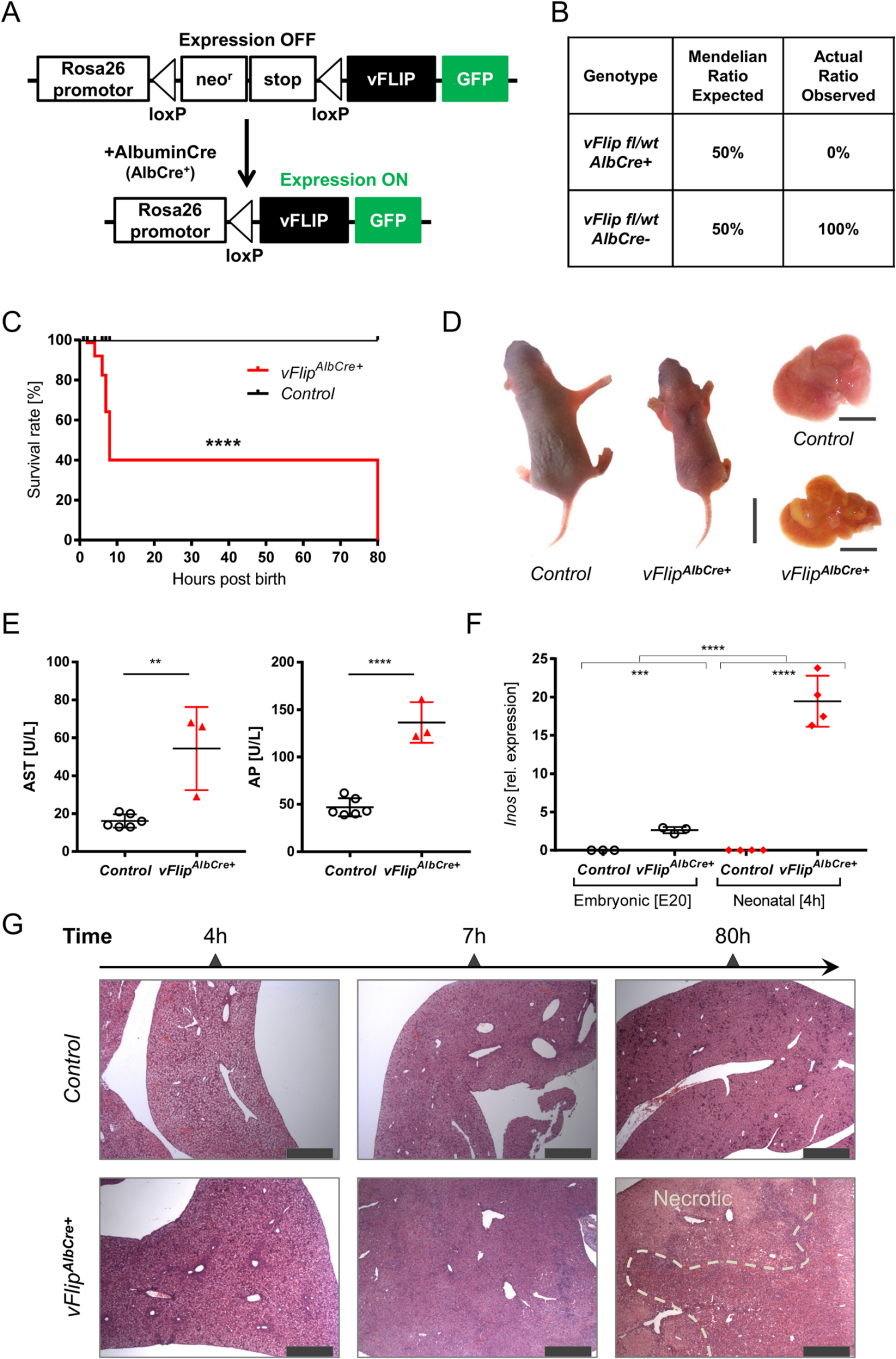
Transgenic expression of *vFlip* in hepatocytes induces severe liver damage. **a** Strategy for generating *vFlip*^*AlbCre+*^ mice. **b** Percentage of viable mice per genotype observed at 3-weeks of age in correlation to the expected Mendelian ratio. **c** Kaplan-Meyer survival analysis of *vFlip*^*AlbCre+*^ (*n* = 11) versus control (*n* = 13) mice starting from birth. **d**–**g** Analysis of *vFlip*^*AlbCre+*^ mice and control littermates. **d** Picture of neonates and neonatal liver (80 h). Scale bar: 10 mm and 5 mm. **e** Serum Aspartate aminotransferase (AST) and Alkaline Phosphatase (AP) concentration. **f** Quantification of relative mRNA expression of *Inos* in the liver of embryonal (E20; *n* = 3/group) and neonatal (4 h post birth; *n* = 4/group) mice. **g** Representative H&E stainings of liver cross-sections at indicated time points post birth. Scale bar 500 µm. Gene expression levels are shown relative to *Hprt*. Error bars indicate ± SD, **P < 0.01, ***P < 0.001, ****P < 0.0001 by unpaired two-tailed t-test, intergroup comparison was performed via one-way ANOVA analysis.

In order to investigate the impact of vFLIP on the host-cell death machinery in the liver, we generated mice expressing *vFlip* in hepatocytes. Rosa26.*vFlip* mice were crossed to mice expressing Cre Recombinase under the control of the hepatocyte-specific Albumin promoter (AlbCre) (Fig. 1a). Surprisingly, no mice expressing *vFlip* in the liver (*vFlip*^*AlbCre+*^) could be obtained post weaning (Fig. 1b). To elucidate, if transgenic *vFlip* expression might already adversely affect embryonic or neonatal liver development leading to an early lethality of transgenic mice, we analyzed embryos one day prior birth (E20) and neonates 4 h post birth. Interestingly, at both time points *vFlip*^*AlbCre+*^ mice were still present at expected 1:1 Mendelian ratio. Therefore, we monitored litters starting from birth (0 h) to identify the timepoint for the loss of *vFlip*^*AlbCre+*^ mice. Surprisingly, we observed that transgenic expression of *vFlip* led to perinatal lethality within the first days post birth. Within 8 h post birth nearly 75% of all analysed *vFlip*^*AlbCre+*^ mice died and only a few survived till day 3 (Fig. 1c), which exhibited reduced body size and altered skin color (Fig. 1d), indicating a developmental delay compared to control mice. Moreover, we observed severe pathological liver changes such as yellowish discoloration and white patches as signs for necrotic tissue (Fig. 1d). Liver damage was further confirmed by significantly increased serum levels of aspartate aminotransferase (AST) and alkaline phosphatase (AP) (Fig. 1e) of transgenic mice, indicating that *vFlip* expression compromises neonatal development of the liver.

Further, we wanted to elucidate, if the onset and increase of *vFlip* expression correlates with perinatal lethality of transgenic mice. Since *vFlip* expression is not directly measurable, we analyzed the level of Alb-Cre Recombinase activity from *tdTomato*^*AlbCre+*^ mice, which produce a red fluorescence protein (tdTomato) instead of vFLIP in cells proportional to Cre Recombinase activity. We compared the signal intensity from embryonic (E20) and neonatal (7 h post birth) *tdTomato*^*AlbCre+*^ liver cryosections. Indeed, tdTomato signal was already detectable at a low level throughout the embryonic liver, but increased significantly post birth (7 h) (Suppl. Fig. 1a). Aligning with this data, we were able to verify the presence of IRES-GFP-vFLIP protein in neonates via western blot (Suppl. Fig. 1b). Therefore, we concluded that there is a correlation between the increasing level of *vFlip* expression and the demise of mice over time.

### Impact of vFLIP activity on liver homeostasis

Based on this, we aimed to characterize the functional activity of vFLIP and its impact on liver homeostasis during perinatal development. Previously, we demonstrated that vFLIP can function as an activator for both the canonical and alternative NF-κB-signaling in vivo^23^. Accordingly, we investigated the expression of the NF-κB target gene *Inos*, as marker for vFLIP functionality and *Albumin*, as marker for liver homeostasis in the embryonic (E20) and neonatal (4 h) liver. *Inos* mRNA expression was significantly increased in neonates expressing *vFlip* (Fig. 1f). Interestingly, we also observed an elevated expression level of *Inos* during embryonic development of *vFlip*^*AlbCre+*^ mice indicating that *vFlip* is already expressed and functionally active during the embryonic stage (Fig. 1f). Despite of vFLIP functionality in embryonal stage, transcriptional level of *Albumin* did not show any significant differences. Whereas, in neonates *Albumin* expression was strongly diminished in *vFlip*^*AlbCre+*^ in comparison to control littermates, suggesting that vFLIP already severely impairs hepatocyte homeostasis within hours post birth (Suppl. Fig. 1c). This result could be confirmed via histopathological analyses revealing no visible differences in tissue morphology or occurrence of cell death between *vFlip*^*AlbCre+*^ mice and control littermates prior to birth (Suppl. Fig. 1d). In contrast, time course dependent histological analysis in neonatal mice uncovered that hepatic *vFlip* expression was accompanied by disturbance of tissue homeostasis through induction of extensive liver damage starting from 4 h post birth (Fig. 1g). Already 7 h post birth we observed extensive hepatocyte degeneration, lack of veins and sinusoids as well as tremendous liver damage as final stage in liver injury (Fig. 1g). In line with the necrotic morphology of dying hepatocytes and the associated tissue damage, we observed increased mRNA transcripts of inflammatory markers such as *Tnf* and *S100a9* (Suppl. Fig. 1e). These data suggest that expression of a single viral protein in hepatocytes is sufficient to cause severe liver injury that promotes an early neonatal death of mice.

### *vFlip* expression triggers hepatocellular necrosis

**Fig. 2:**
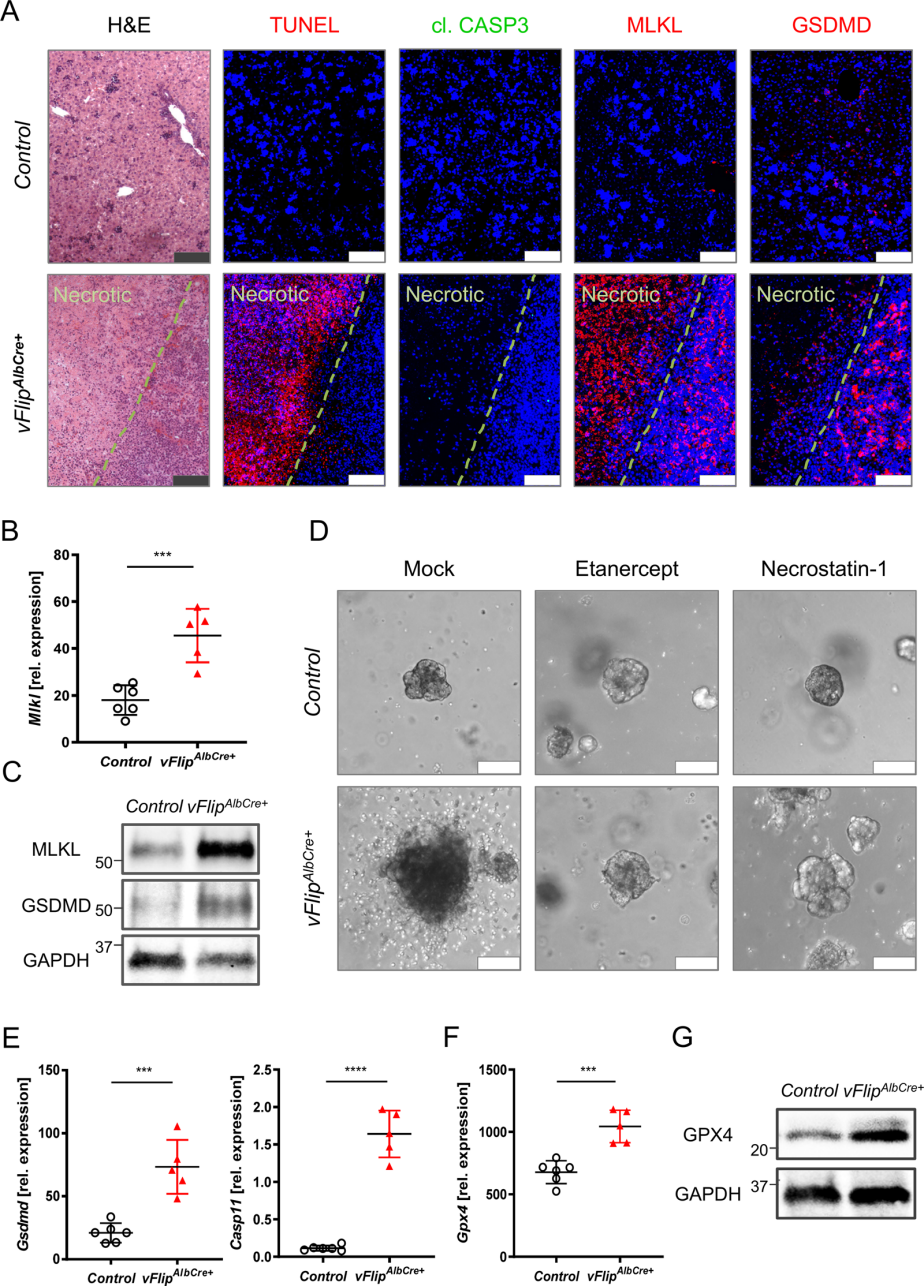
Hepatic *vFlip* expression triggers regulated necrosis in hepatocytes. **a**–**e** Representative data derived from neonatal liver tissue of *vFlip*^*AlbCre+*^ (*n* = 5) and control littermates (*n* = 6). Experiments were repeated three times with similar results. **a** Representative stainings of H&E, TUNEL (red), cl. CASP3 (green), MLKL (red), GSDMD (red) counterstained with Hoechst (blue, nuclei) in consecutive liver cross-sections. Scale bar: 100 µm. **b** Relative mRNA expression of *Mlkl* in the liver. **c** Detection of hepatic MLKL and GSDMD protein level by Western Blot. GAPDH was used as loading control. **d** Representative images via z-stack confocal microscopy from mock, Etanercept (TNF inhibitor) and Necrostatin-1 (RIPK1 inhibitor) treated liver organoids derived from embryonic *vFlip*^*AlbCre+*^ and control mice. Scale bar: 100 µm. **e**, **f** Relative mRNA expression of genes involved in **e** pyroptosis and **f** lipid peroxidation in the liver of indicated mice. **g** Detection of GPX4 protein level by Western Blot. GAPDH was used as loading control. Gene expression levels are shown relative to *Hprt*. Error bars indicate ± SD, ***P < 0.001, ****P < 0.0001 by unpaired two-tailed *t*-test.

Our data revealed that vFLIP promotes excessive hepatocyte degeneration and massive liver injury. Since vFLIP has been described as a negative regulator of Caspase-8, we next investigated whether liver injury as observed in *vFlip*^*AlbCre+*^ mice was caused by apoptosis or necrosis. Immunohistochemical analyses of consecutive tissue cross-sections exhibited no activation of cleaved CASP3 in areas of severe hepatocellular death, indicated by TUNEL staining (Fig. 2a). In line with these results, we could not observe differences in the mRNA expression level of Caspase-8 (*Casp8*) or its cellular inhibitor cFLIP (*Cflar*), both involved in apoptosis (Suppl. Fig. 2a). These data suggest that hepatocellular necrosis rather than apoptotic cell death contributes to vFLIP induced liver injury. In order to screen for potential molecular mechanisms underlying the observed hepatocellular necrosis, we performed gene expression analysis of key mediators of regulated necrosis. Indeed, we observed elevated mRNA expression (Fig. 2b) and protein levels (Fig. 2c, Suppl. Fig. 2b) of MLKL, an effector protein in the canonical and non-canonical necroptosis pathway, in *vFlip*^*AlbCre+*^ mice. Of note, *Ripk3 a* key element in the formation of the necrosome did not show significant changes (Suppl. Fig. 2c). Since TNF is known as main driver of necroptosis in the absence of caspase-8 activity (i.e., inhibition by vFLIP), we next investigated whether autocrine TNF production in *vFlip* expressing hepatocytes might trigger MLKL-dependent cell death. Therefore, we quantified *Tnf* and *Mlkl* mRNA expression level in liver organoids generated from embryonic *vFlip*^*AlbCre+*^ mice and controls. Indeed, both marker genes were significantly upregulated in *vFlip*^*AlbCre+*^ liver organoids (Suppl. Fig. 2d) indicating that an autocrine TNF production might drive necroptosis in *vFlip*^*AlbCre+*^ hepatocytes. In line with this hypothesis, we observed an increased TNF concentration in the supernatant of liver organoids (ex vivo) (Suppl. Fig. 2e) as well as in neonatal serum (in vivo) derived from *vFlip*^*AlbCre+*^ mice (Suppl. Fig. 2f). In a functional approach, we were able to restore vitality of *vFlip*^*AlbCre+*^ liver organoids by addition of Etanercept, a TNF inhibitor, as well as Necrostatin-1, a RIPK1 inhibitor, supporting the hypothesis that autocrine TNF production triggers MLKL-RIPK1 mediated necroptosis in *vFlip*^*AlbCre+*^ mice (Fig. 2d).

Interestingly, also *Gsdmd*, effector protein of pyroptosis, was significantly upregulated on mRNA and protein level in *vFlip*^*AlbCre+*^ liver (Fig. 2e, +c). To further define upstream signaling cascade of pyroptosis, we examined mRNA expression of Caspase-1 (canonical) and Caspase-11 (non-canonical)^24^. We observed that hepatic *Casp1* gene expression did not show significant differences (Suppl. Fig. 2g), while *Casp11*, which is part of the non-canonical pathway, was significantly increased in livers of transgenic mice (Fig. 2e). To better visualize the localization of necroptosis and pyroptosis mediator proteins in the liver of transgenic mice, we performed immunohistochemical stainings on consecutive tissue sections. Remarkably, signal for MLKL was most pronounced in areas of severe hepatocellular necrosis as indicated by TUNEL-positivity, while GSDMD signal was particularly localized in hepatocytes in adjacent, non-necrotic tissue (Fig. 2a).

Since peroxidated membrane lipids play a critical role in the pore formation process of both MLKL and GSDMD^25,26^, and *Casp11* expression can be induced by lipid peroxidation^26^, we were interested how expression of selected marker genes involved in oxidative stress management would be effected under *vFlip* expression in the liver. Indeed, we observed elevated mRNA expression of *Gpx4* (Fig. 2f)*, Acsl4*, and *Slc7a11* (Suppl. Fig. 2h), as well as an increased protein level for GPX4 (Fig. 2g), which is typically upregulated in liver tissue upon lipid peroxidation and postulated as inhibitor for the activation of Caspase-11 and Caspase-1^26^.

Together these findings suggest that the presence of the viral caspase-8 inhibitor vFLIP triggers hepatocellular necrosis via a RIPK1-MLKL-dependent pathway driven by an autocrine TNF production accompanied by the upregulation of marker genes for lipid peroxidation and pyroptosis.

### Impact of vFLIP induced hepatocellular necrosis on bile acid biosynthesis and transport

**Fig. 3:**
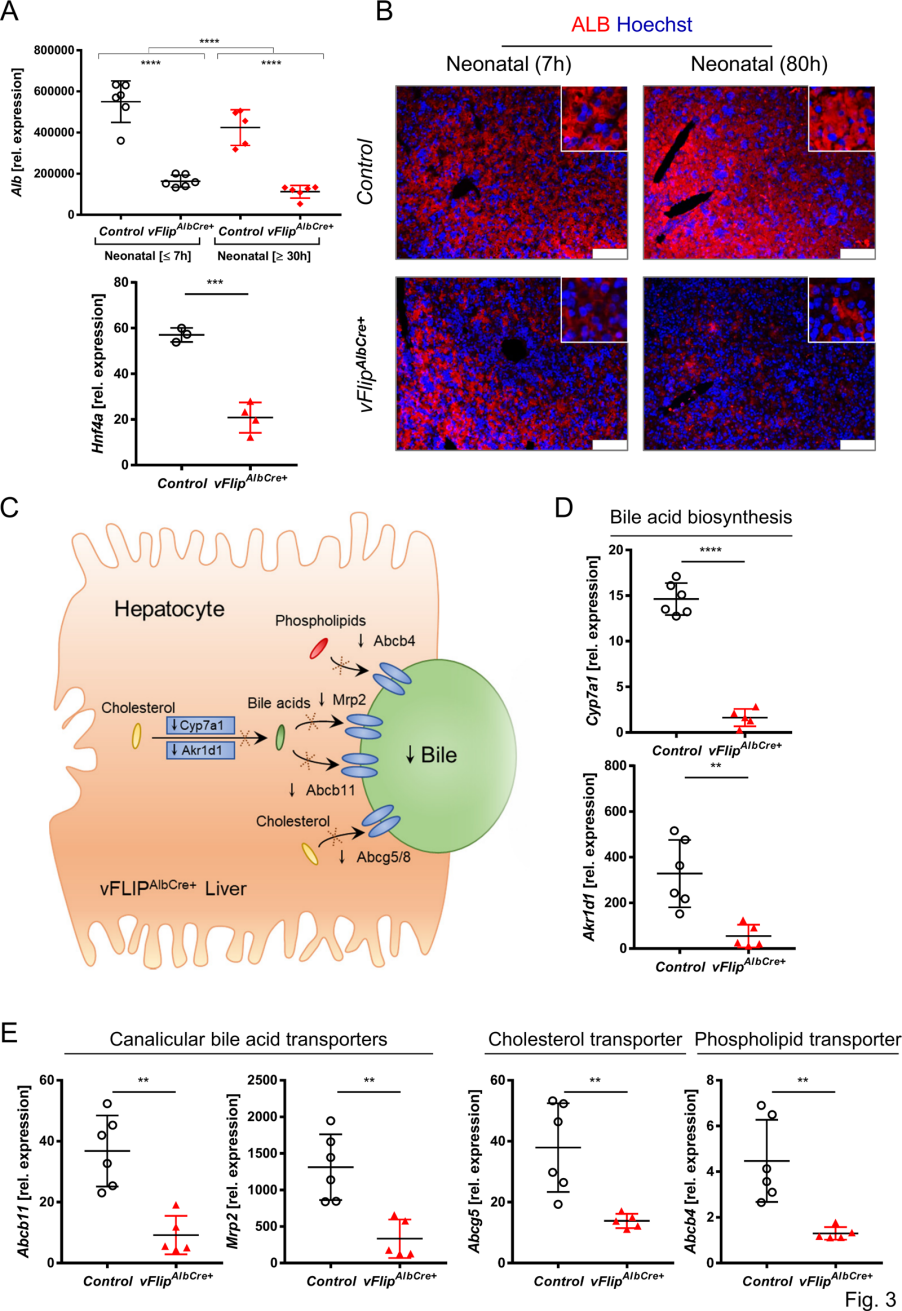
Impact of hepatic *vFlip* expression on bile acid biosynthesis and transport. **a**–**b** Representative data derived from neonatal liver tissue of *vFlip*^*AlbCre+*^ and control littermates at ≤7 h and ≥30 h post birth. Experiments were repeated 3 times with similar results. **a** Relative mRNA expression of indicated genes. **b** Immunohistochemical stainings from liver cross-sections stained for Albumin (ALB, red; hepatocytes) counterstained with Hoechst (blue, nuclei). Scale bar 100 µm. **c** Simplified schematic overview of key elements in bile production and transport affected in *vFlip*^*AlbCre+*^ mice. **d**–**e** Representative data derived from neonatal liver tissue of *vFlip*^*AlbCre+*^ (*n* = 5) and control littermates (*n* = 6). Experiments were repeated 3 times with similar results. Relative mRNA expression of genes involved in **d** bile acid biosynthesis and **e** bile transport in the liver of indicated mice. Gene expression levels are shown relative to *Hprt*. Error bars indicate ± SD, **P < 0.01, ***P < 0.001, ****P < 0.0001 by unpaired two-tailed t-test, intergroup comparison was performed via one-way ANOVA analysis.

After characterizing different forms of cell death involved in hepatocellular necrosis in *vFlip*^*AlbCre+*^ mice, we were further interested how *vFlip* expression impacts liver homeostasis. Therefore, we analyzed several marker genes expressed by hepatocytes on transcriptional level. Within several hours *Albumin* and *Hnf4a* mRNA transcripts were already significantly reduced in *vFlip*^*AlbCre+*^ compared to control neonates (Fig. 3a). Immunohistochemical staining confirmed a decrease in Albumin signal in the *vFlip*^*AlbCre+*^ liver (80 h post birth) (Fig. 3b), suggesting a drastic loss of hepatocytes over time.

Hepatocytes play a critical role in bile acid biosynthesis, metabolism and transport^27^ (Fig. 3c). Accordingly, hepatocyte dysfunction can lead to impaired bile acid production and reduced transport. Indeed, hepatic expression of *Cyp7a1* and *Akr1d1*, key enzymes in catalyzing bile acid biosynthesis from cholesterol were severely downregulated in *vFlip*^*AlbCre+*^ mice (Fig. 3d). Also the transcriptional level for essential export pumps in the hepatobiliary transport systems such as *Abcb11*, *Mrp2* for bile acids, *Abcg5* for cholesterol as well as *Abcb4* for phosphatidylcholine were decreased (Fig. 3e)^28^. In summary, these data suggest that *vFlip*^*AlbCre+*^ mice show loss of hepatocyte functionality resulting in defective bile acid biosynthesis and transport.

### Transgenic expression of *vFlip* in hepatocytes affects homeostasis of cholangiocytes

**Fig. 4:**
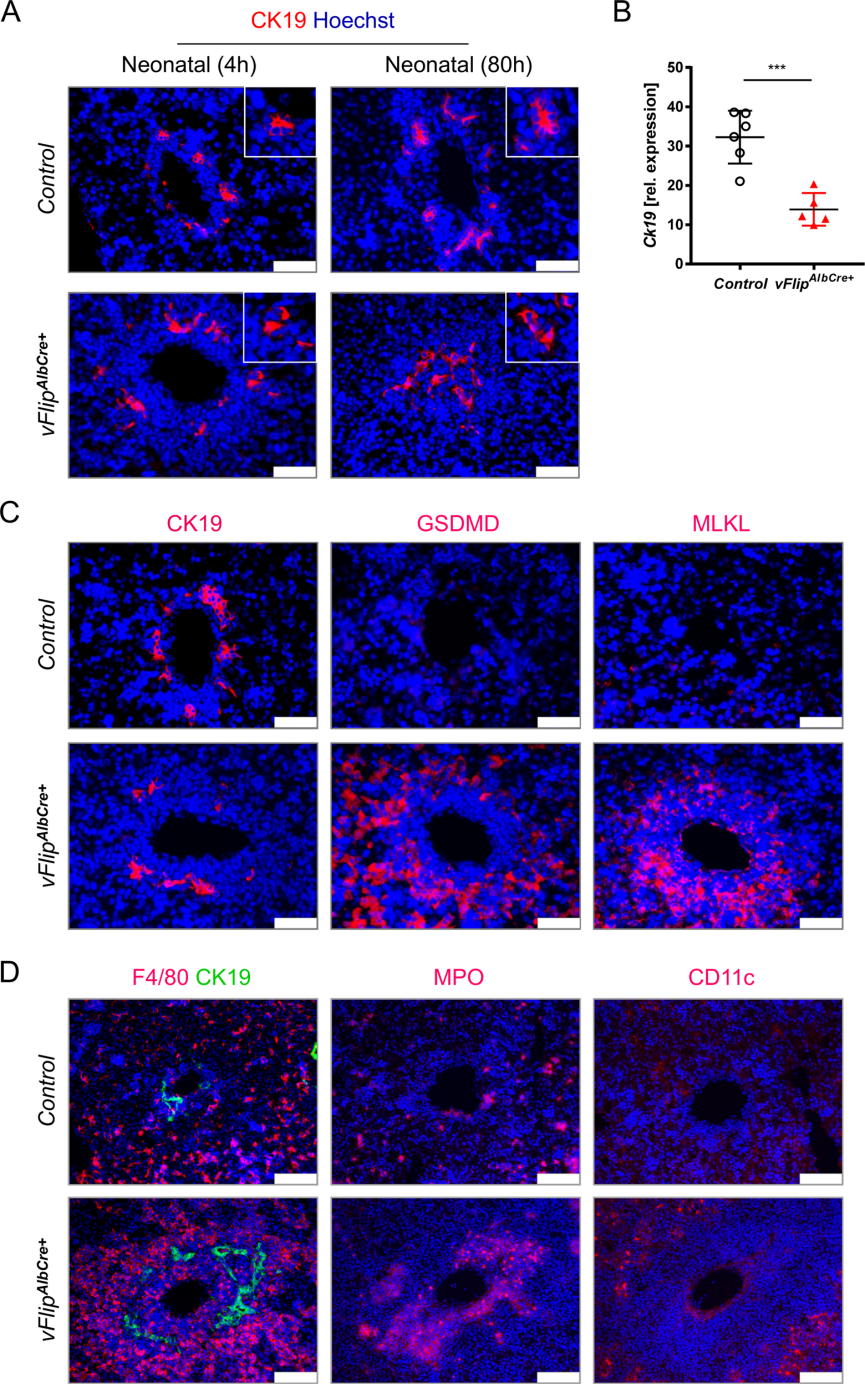
Effect of hepatocellular necrosis on the biliary system. **a**–**c** Representative data derived from neonatal liver tissue of *vFlip*^*AlbCre+*^ (*n* = 5) and control littermates (*n* = 6). Experiments were repeated 3 times with similar results. **a** Immunohistochemical staining of cytokeratin-19 (CK19, red) counterstained with Hoechst (blue, nuclei) in liver cross-sections of control or *vFlip*^AlbCre+^ mice at early (4 h p.b.) and late (80 h p.b.) time points. Scale bar 50 µm. **b** Relative expression of *Ck19* in liver tissue. **c** Representative stainings of CK19 (red), MLKL (red), GSDMD (red) counterstained with Hoechst (blue, nuclei) in consecutive liver cross-sections. Scale bar 50 µm. **d** Representative images of immunohistochemical stainings of cholangiocytes (CK19), Kupffer cells (F4/80), neutrophils (MPO), and dendritic cells (CD11c) in liver cross-sections of *vFlip*^*AlbCre+*^ and control mice. Nucleic counterstaining with Hoechst (blue). Scale bar: 250 µm. Gene expression levels are shown relative to *Hprt*. Error bars indicate ± SD, ***P < 0.001 by unpaired two-tailed *t*-test.

Cholangiocytes, the lining epithelial cells in the three-dimensional network of bile ducts, play a crucial role in the transport of bile to the gut^29^. Therefore, we were interested, if impaired bile production and transport in transgenic mice would have a secondary effect on cholangiocyte homeostasis. Interestingly, immunohistochemical staining of Cytokeratin-19 (CK19) uncovered progressing structural deformation of cholangiocytes over time in transgenic mice (Fig. 4a), culminating in the loss of cholangiocytes, confirmed by reduced Cytokeratin-19 gene expression (Fig. 4b) and protein level (Suppl. Fig. 3). Furthermore, we uncovered that GSDMD and MLKL signal was strong in the area of damaged bile ducts surrounding the portal vein (Fig. 4c), which was accompanied by distinct immune cell accumulations (Fig. 4d).

In summary these data suggest that expression of *vFlip* in hepatocytes is accompanied by impaired bile production and transport, culminating in deformation and loss of bile ducts.

### Hepatocellular necrosis affects intestinal homeostasis

**Fig. 5:**
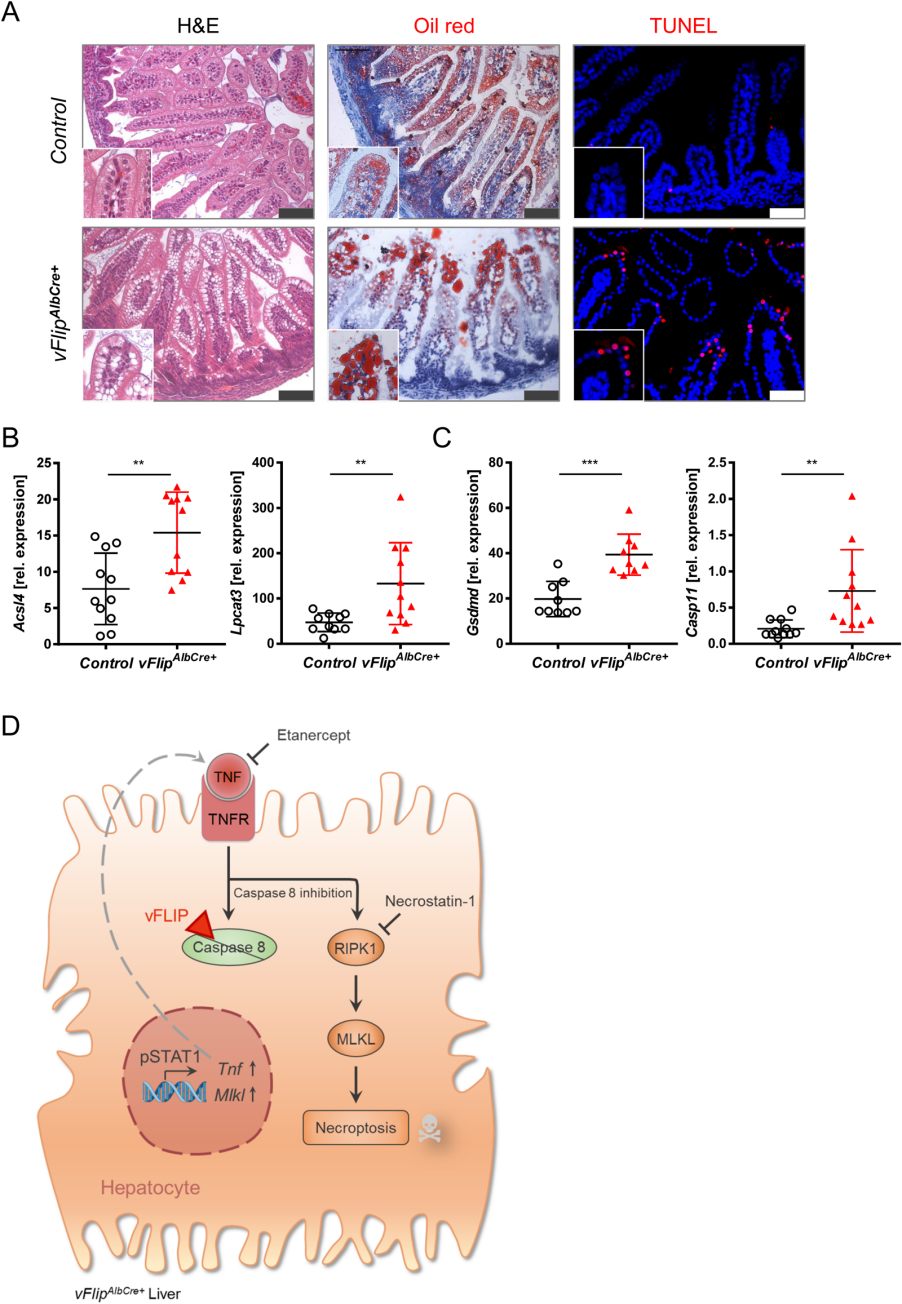
Effect of liver necrosis and defective bile acid production on intestinal homeostasis. **a**–**c** Representative data derived from neonatal duodenum of *vFlip*^*AlbCre+*^ (*n* = 5) and control littermates (*n* = 6). Experiments were repeated three times with similar results. **a** Representative stainings from duodenum cross-sections stained with H&E, OilRed (red, lipids) and TUNEL assay (red) counterstained with Hoechst (blue, nuclei). Scale bar 100 µm. **b**, **c** Relative mRNA expression of genes involved in **b** lipid peroxidation and **c** pyroptosis in the liver of indicated mice. **d** Simplified schematic overview of vFLIP induced regulated necrosis in the liver. vFLIP: viral Fas-associated death domain-like interleukin-1β-converting enzyme-inhibitory protein, TNF: Tumor necrosis factor, TNFR: TNF receptor, RIPK1: Receptor-interacting protein kinase 1, MLKL: Mixed lineage kinase domain-like pseudokinase. Gene expression levels are shown relative to *Hprt*. Error bars indicate ± SD, **P < 0.01, ***P < 0.001 by unpaired two-tailed *t*-test.

Since bile acids are transported via the biliary system to the gut and are essential for the absorption of dietary fats and vitamins^30^, impaired bile acid metabolism may not only impact liver and bile ducts, but also potentially intestinal lipid absorption of *vFlip*^*AlbCre-+*^ mice. Indeed, we observed droplet formation in the upper villus tips of the duodenal epithelium of transgenic neonates (Fig. 5a). Oil red staining identified these droplets as lipid accumulations (Fig. 5a). Since strong lipid accumulation potentially increases the risk for lipid peroxidation and recent publications have shown the involvement of lipid peroxidation in pyroptosis mediated cell death^26^, we were interested how the observed lipid accumulation might influence cell death in the intestine of *vFlip*^*AlbCre-+*^ mice. Indeed, we could identify TUNEL positive pycnotic nuclei of dead cells localized particularly at duodenal villus tips of *vFlip*^*AlbCre+*^ mice (Fig. 5a). We further recorded increased gene expression levels of mediators in lipid peroxidation (*Acsl4, Lpcat3*) (Fig. 5b), as well as pyroptosis (*Gsdmd*, *Casp11*) (Fig. 5c) in the duodenum of transgenic mice. Of note, no other organ (including lung and kidney) except for liver and gut exhibited pathological features or signs of cell death (Suppl. Fig. 4a, b).

## Discussion

The liver plays an important role in host defense against pathogenic microorganisms. Especially the invasion of opportunistic viruses can lead to a wide variety of pathological manifestations^31^. Although it is known that many of these viruses produce proteins actively interfering with the host-cell death machinery^32^, there is still only limited mechanistical knowledge available. In this study we investigated consequences of constitutive expression of vFLIP, a Caspase-8 inhibitor^20,23,33,34^, in hepatic epithelial cells. Expression of this viral protein was sufficient to induce histopathological features of acute viral hepatitis (AVH) and acute-on-chronic liver failure (ACLF) with extended areas of hepatocellular necrosis associated with severe loss of hepatocytes^35,36^. At a molecular level, we uncovered that hepatocellular death in *vFlip*^*AlbCre+*^ mice was mediated by regulated necrosis, leading to impaired liver homeostasis including compromised bile acid biosynthesis and transport. In summary, our data indicate that expression of a viral apoptosis inhibitor has the potential to induce hepatocellular necrosis.

Caspase-8 has been described as a decisive ‘checkpoint’ in anti-viral response due to its function as key switch between apoptosis, necroptosis and cell survival^10–15,37,38^. Therefore, many viruses have developed different strategies to inhibit Caspase-8 activation^16^ including expression of viral proteins directly binding and blocking pro-caspase-8 (e.g., viral FLIP^39–41^ from KSHV^17,42^, v-ICA from hCMV^43^) or upregulation of cellular Caspase-8 inhibitors such as cFLIP (e.g., Core protein from HCV^44^ and LMP1 from EBV^45^). Given the abundance of viruses in the liver, it may be reasoned that during infection viruses expressing cell death regulating proteins such as vFLIP^46–48^ might trigger non-apoptotic programmed cell death associated with hepatic inflammation. Indeed, several viruses encoding anti-apoptotic proteins, such as hCMV and EBV, have been identified to play a significant role in patients with AVH and less frequently in ACLF^49^.

Apoptosis inhibitory function of vFLIP has been demonstrated in vitro in several cell lines^17,41,46^. In accordance with these studies, we observed that transgenic vFlip expression not only blocks apoptosis but also induces massive regulated necrosis with typical features of necroptosis. This supports the hypothesis that regulated necrosis might function as a backup mechanism to enable cell death in settings where apoptosis is inhibited by e.g. viruses^50^. Furthermore, recent publications suggest that viruses also express inhibitors for regulated necrosis primarily targeting RIPK3^51,52^. Interestingly, we have recently uncovered a RIPK3-independent form of necroptosis in the context of hepatic disease^53^. In line with these results, we identified via functional analyses that necroptosis was mediated via a RIPK1-MLKL-dependent pathway, in the *vFlip*^*AlbCre+*^ liver, suggesting further evolutionary adaption processes of the liver to recurrent invasion and persistence of viruses.

Further, we identified an upregulation of cell death effector proteins in and in close proximity to the necrotic tissue. Particularly, MLKL expression was strongly induced in necrotic areas, while GSDMD positive cells were localized in the adjacent tissue. Similar effects have been observed in recent in vitro HCV infection studies, where uninfected neighboring cells underwent pyroptosis^54^. In addition to the increased levels of GSDMD in transgenic liver tissue we identified an upregulation of marker genes involved in non-canonical pyroptosis as well as lipid peroxidation. Interestingly, a very recent study uncovered that GPX4 coordinates lipid peroxidation-dependent Caspase-11 activation promoting GSDMD cleavage and thus pyroptosis^26^. These data suggest a correlation in the upregulation of these different cell death pathways, as seen in *vFlip*^*AlbCre+*^ mice, with a jet unknown molecular mechanism. The interplay of different forms of regulated necrosis in the context of viral cell death regulation in infected livers has to be further evaluated.

In conclusion, our findings suggest that transgenic expression of a single viral protein can be sufficient to induce hepatocellular necrosis and acute liver failure. Accordingly, our study might contribute to a more profound understanding of the molecular mechanism of host-cell death responses to viral infection in the liver.

## Supplementary information

Supplementary Figure Legends

Suppl. Fig. 1

Suppl. Fig. 2

Suppl. Fig. 3

Suppl. Fig. 4

## References

[1] Morissette G, Flamand L (2010). Herpesviruses and chromosomal integration. J. Virol..

[2] Zimmermann HW, Trautwein C, Tacke F (2012). Functional role of monocytes and macrophages for the inflammatory response in acute liver injury. Front. Physiol..

[3] Chen Y, Williams V, Filippova M, Filippov V, Duerksen-Hughes P (2014). Viral carcinogenesis: factors inducing DNA damage and virus integration. Cancers.

[4] Luedde T, Kaplowitz N, Schwabe RF (2014). Cell death and cell death responses in liver disease: mechanisms and clinical relevance. Gastroenterology.

[5] Matzinger P (2002). The danger model: a renewed sense of self. Science.

[6] Rock KL, Jiann-Jyh L, Hajime K (2011). Innate and adaptive immune responses to cell death. Immunol. Rev..

[7] Canbay A, Friedman S, Gores GJ (2004). Apoptosis: the nexus of liver injury and fibrosis. Hepatology.

[8] Vanden Berghe T, Linkermann A, Jouan-Lanhouet S, Walczak H, Vandenabeele P (2014). Regulated necrosis: the expanding network of non-apoptotic cell death pathways. Nat. Rev. Mol. Cell Biol..

[9] Galluzzi L (2018). Molecular mechanisms of cell death: recommendations of the Nomenclature Committee on Cell Death 2018. Cell Death Differ..

[10] Stennicke HR (1998). Pro-caspase-3 is a major physiologic target of caspase-8. J. Biol. Chem..

[11] Huang K (2016). Cleavage by caspase 8 and mitochondrial membrane association activate the BH3-only protein bid during TRAIL-induced. J. Biol. Chem..

[12] Ng FW, Shore GC (1998). Bcl-XL cooperatively associates with the Bap31 complex in the endoplasmic reticulum, dependent on procaspase-8 and Ced-4 adaptor. J. Biol. Chem..

[13] Ng FWH (1997). p28 Bap31, a Bcl-2/Bcl-X(L)- and procaspase-8–associated protein in the endoplasmic reticulum. J. Cell Biol..

[14] Wang B (2003). Uncleaved BAP31 in association with A4 protein at the endoplasmic reticulum is an inhibitor of Fas-initiated release of cytochrome c from mitochondria. J. Biol. Chem..

[15] Breckenridge DG, Stojanovic M, Marcellus RC, Shore GC (2003). Caspase cleavage product of BAP31 induces mitochondrial fission through endoplasmic reticulum calcium signals, enhancing cytochrome c release to the cytosol. J. Cell Biol..

[16] Galluzzi L (2010). Viral strategies for the evasion of immunogenic cell death. J. Intern. Med..

[17] Belanger C (2001). Human herpesvirus 8 viral FLICE-inhibitory protein inhibits Fas-mediated apoptosis through binding and prevention of procaspase-8 maturation. J. Hum. Virol..

[18] Kim B, Jeon YK, Kim CW (2009). Kaposi sarcoma herpes virus-associated hemophagocytic syndrome complicated by multicentric castleman disease and kaposi sarcoma in a HIV-negative immunocompetent patient: an autopsy case. J. Korean Med. Sci..

[19] Kataoka T (2000). The caspase-8 inhibitor FLIP promotes activation of NF-kappaB and Erk signaling pathways. Curr. Biol..

[20] Ballon G, Chen K, Perez R, Tam W, Cesarman E (2011). Kaposi sarcoma herpesvirus (KSHV) vFLIP oncoprotein induces B cell transdifferentiation and tumorigenesis in mice. J. Clin. Invest..

[21] Madisen L (2010). A robust and high-throughput Cre reporting and characterization system for the whole mouse brain. Nat. Neurosci..

[22] Lillie RD, Ashburn LL (1943). Supersaturated solutions of fat stains in dilute isopropanol for demonstration of acute fatty degeneration not shown by Herxheimer’s technique. Archs. Path..

[23] Ruder B (2018). Chronic intestinal inflammation in mice expressing viral Flip in epithelial cells. Mucosal Immunol..

[24] Sborgi L (2016). GSDMD membrane pore formation constitutes the mechanism of pyroptotic cell death. EMBO J..

[25] Magtanong L, Ko PJ, Dixon SJ (2016). Emerging roles for lipids in non-apoptotic cell death. Cell Death Differ..

[26] Kang R (2018). Lipid peroxidation drives gasdermin D-mediated pyroptosis in lethal polymicrobial sepsis. Cell Host Microbe.

[27] Chiang JYL (2017). Bile acid metabolism and signaling in liver disease and therapy. Liver Res..

[28] Geier A, Fickert P, Trauner M (2006). Mechanisms of disease: mechanisms and clinical implications of cholestasis in sepsis. Nat. Clin. Pract. Gastr..

[29] Tabibian, J. H., Masyuk, A. I., Masyuk, T. V., O’Hara, S. P. & LaRusso, N. F. Physiology of cholangiocytes. *Compr. Physiol*. 10.1002/cphy.c120019 (2013). 10.1002/cphy.c120019 PMC3831353 23720296

[30] Xia X, Francis H, Glaser S, Alpini G, LeSage G (2006). Bile acid interactions with cholangiocytes. World J. Gastroenterol..

[31] Talwani R, Gilliam BL, Howell C (2011). Infectious diseases and the liver. Clin. Liver Dis..

[32] Kaminskyy V, Zhivotovsky B (2010). To kill or be killed: how viruses interact with the cell death machinery. J. Intern. Med..

[33] Ballon G, Akar G, Cesarman E (2015). Systemic expression of Kaposi sarcoma herpesvirus (KSHV) Vflip in endothelial cells leads to a profound proinflammatory phenotype and myeloid lineage remodeling in vivo. PLoS Pathog..

[34] Glykofrydes D (2000). Herpesvirus Saimiri vFLIP provides an antiapoptotic function but is not essential for viral replication, transformation, or pathogenicity. J. Virol..

[35] Krishna M (2017). Patterns of necrosis in liver disease. Clin. Liver Dis. (Hoboken).

[36] Kim H, Park YN (2010). Massive hepatic necrosis with large regenerative nodules. Korean J. Hepatol..

[37] Panaretakis T (2009). Mechanisms of pre-apoptotic calreticulin exposure in immunogenic cell death. EMBO.

[38] Fischer U, Stroh C, Schulze-Osthoff K (2006). Unique and overlapping substrate specificities of caspase-8 and caspase-10. Oncogene.

[39] Bertin J (1997). Death effector domain-containing herpesvirus and poxvirus proteins inhibit both Fas- and TNFR1-induced apoptosis. Proc. Natl Acad. Sci. USA.

[40] Hu S, Vincenz C, Buller M, Dixit VM (1997). A novel family of viral death effector domain-containing molecules that inhibit both CD-95- and tumor necrosis factor receptor-1-induced apoptosis. J. Biol. Chem..

[41] Thome M (1997). Viral FLICE-inhibitory proteins (FLIPs) prevent apoptosis induced by death receptors. Nature.

[42] Stürzl M (1999). Expression of K13/v-FLIP gene of human herpesvirus 8 and apoptosis in Kaposi’s sarcoma spindle cells. J. Natl Cancer Inst..

[43] Skaletskaya A (2001). A cytomegalovirus-encoded inhibitor of apoptosis that suppresses caspase-8 activation. Proc. Natl Acad. Sci. USA.

[44] Saito K (2006). Hepatitis C virus core protein inhibits tumor necrosis factor alpha-mediated apoptosis by a protective effect involving cellular FLICE inhibitory protein. J. Virol..

[45] Snow AL (2006). EBV can protect latently infected B cell lymphomas from death receptor-induced apoptosis. J. Immunol..

[46] Sun Q, Matta H, Chaudhary PM (2003). The human herpes virus 8–encoded viral FLICE inhibitory protein protects against growth factor withdrawal–induced apoptosis via NF-κB activation. Blood.

[47] Matta H, Chaudhary PM (2004). Activation of alternative NF-κB pathway by human herpes virus 8-encoded Fas-associated death domain-like IL-1β-converting enzyme inhibitory protein (vFLIP). Proc. Natl Acad. Sci. USA.

[48] An J, Sun Y, Sun R, Rettig MB (2003). Kaposi’s sarcoma-associated herpesvirus encoded vFLIP induces cellular IL-6 expression: the role of the NF-kappaB and JNK/AP1 pathways. Oncogene.

[49] Gupta E, Ballani N, Kumar M, Sarin SK (2015). Role of non-hepatotropic viruses in acute sporadic viral hepatitis and acute-on-chronic liver failure in adults. Indian J. Gastroenterol..

[50] Han J, Zhong CQ, Zhang DW (2011). Programmed necrosis: backup to and competitor with apoptosis in the immune system. Nat. Immunol..

[51] Dondelinger Y, Hulpiau P, Saeys Y, Bertrand MJM, Vandenabeele P (2016). An evolutionary perspective on the necroptotic pathway. Trends Cell Biol..

[52] Kearney CJ, Martin SJ (2017). An inflammatory perspective on necroptosis. Mol. Cell.

[53] Gunther C (2016). The pseudokinase MLKL mediates programmed hepatocellular necrosis independently of RIPK3 during hepatitis. J. Clin. Invest..

[54] Kofahi HM, Taylor NGA, Hirasawa K, Grant MD, Russell RS (2016). Hepatitis C virus infection of cultured human hepatoma cells causes apoptosis and pyroptosis in both infected and bystander cells. Sci. Rep..

