# Supplementary material for: Modulation of the extrinsic cell death signaling pathway by viral Flip induces acute-death mediated liver failure
Source: Cell Death Dis. 2019 Nov 21;10(12):878. doi: 10.1038/s41419-019-2115-y (PMC6872756; doi:10.1038/s41419-019-2115-y)
Supplement: Supplementary file 1 — Supplementary Figure Legends [file 41419_2019_2115_MOESM1_ESM.docx]

**Supplementary Figure Legends**

**Suppl. Fig. 1: Characterization of *vFlip* expression *in vivo***

**a** Hoechst counterstained liver cryosections from *tdTomato^AlbCre+^* and control mice prior (E20) and post birth (7h). Cells with active Cre Recombinase express *tdTomato* (red). Scale bar 200µm. **b** Western Blot analysis of GFP tagged vFLIP using liver lysates from indicated mice (asterisk, unspecific band). GAPDH was used as a loading control. **c** Quantification of relative mRNA expression of *Alb* in the liver of embryonic (E20) and neonatal (4h post birth) control and *vFlip^AlbCre+^* mice (n=3/group). **d** Representative images of liver sections stained with H&E or TUNEL assay (red, cell death) counterstained with Hoechst (blue, nuclei) of *vFlip^AlbCre+^* and control littermates prior (E20) and post birth. Scale bar 200µm. **e** mRNA expression level of inflammatory marker genes in the liver of *vFlip^AlbCre+^* and control littermates.

Gene expression levels are shown relative to *Hprt*. Error bars indicate ±SD, ns P ≥ 0.05, ***P* < 0.01, *****P* < 0.0001 by unpaired two-tailed t-test, intergroup comparison was performed via one-way ANOVA analysis.

**Suppl. Fig. 2: Expression of cell death mediators in the liver of *vFlip^AlbCre+^* mice**

**a-c** Representative data derived from neonatal liver tissue of *vFlip^AlbCre+^* (n=5) and control littermates (n=6). Experiments were repeated 3 times with similar results. **a** Real-time PCR analysis of genes related to apoptosis. **b** Immunohistochemical staining with a total MLKL antibody (red, arrows). Nuclei were counterstained with Hoechst (blue). Z-stack pictures were taken via confocal microscopy. Scale bar: 200µm (left), 20µm (right). **c** mRNA expression level of *Ripk3***. d+e** Depicted data derived from liver organoid cultures generated from embryonic *vFlip^AlbCre+^* and control littermates. **d** Relative mRNA expression of *Tnf* and *Mlkl*. **e+f** TNF concentration measured by ELISA **e** from liver organoid culture supernatant and **f** serum from neonatal mice (7h post birth). **g+h** Real-time PCR analysis of genes related to **g** pyroptosis and **h** lipid peroxidation from the liver of *vFlip^AlbCre+^* and control littermates.

Gene expression levels are shown relative to *Hprt*. Error bars indicate ±SD, ns P ≥ 0.05, *****P* < 0.0001 by unpaired two-tailed t-test.

**Suppl. Fig. 3: Cholangiocytes are lost in close proximity to the necrotic tissue in *vFlip^AlbCre+^* mice**

Representative confocal images of TUNEL, CK19 stained liver cross-sections of *vFlipxtdtomato^AlbCre+^* and control littermates. Hoechst (blue, Nuclei), TUNEL (Green, Cell death), CK19 (white, cholangiocytes) tdtomato^AlbCre+^ (red, hepatocytes). Scale bar: 250µm (left), 75µm (right).

**Suppl. Fig. 4: Secondary effect of hepatocellular *vFlip* expression on other organs**

**a+b** Data derived from neonatal *vFlip^AlbCre+^* (n=5) and control littermates (n=6). Experiments were repeated 3 times with similar results. Representative stainings of liver, lung and kidney sections stained with **a** H&E, Scale bar 100 µm; and **b** TUNEL assay (red, cell death) counterstained with Hoechst (blue, nuclei). Scale bar 200 µm.
