# Supplementary figures and images for: Modulation of the extrinsic cell death signaling pathway by viral Flip induces acute-death mediated liver failure
Source: Cell Death Dis. 2019 Nov 21;10(12):878. doi: 10.1038/s41419-019-2115-y (PMC6872756; doi:10.1038/s41419-019-2115-y)

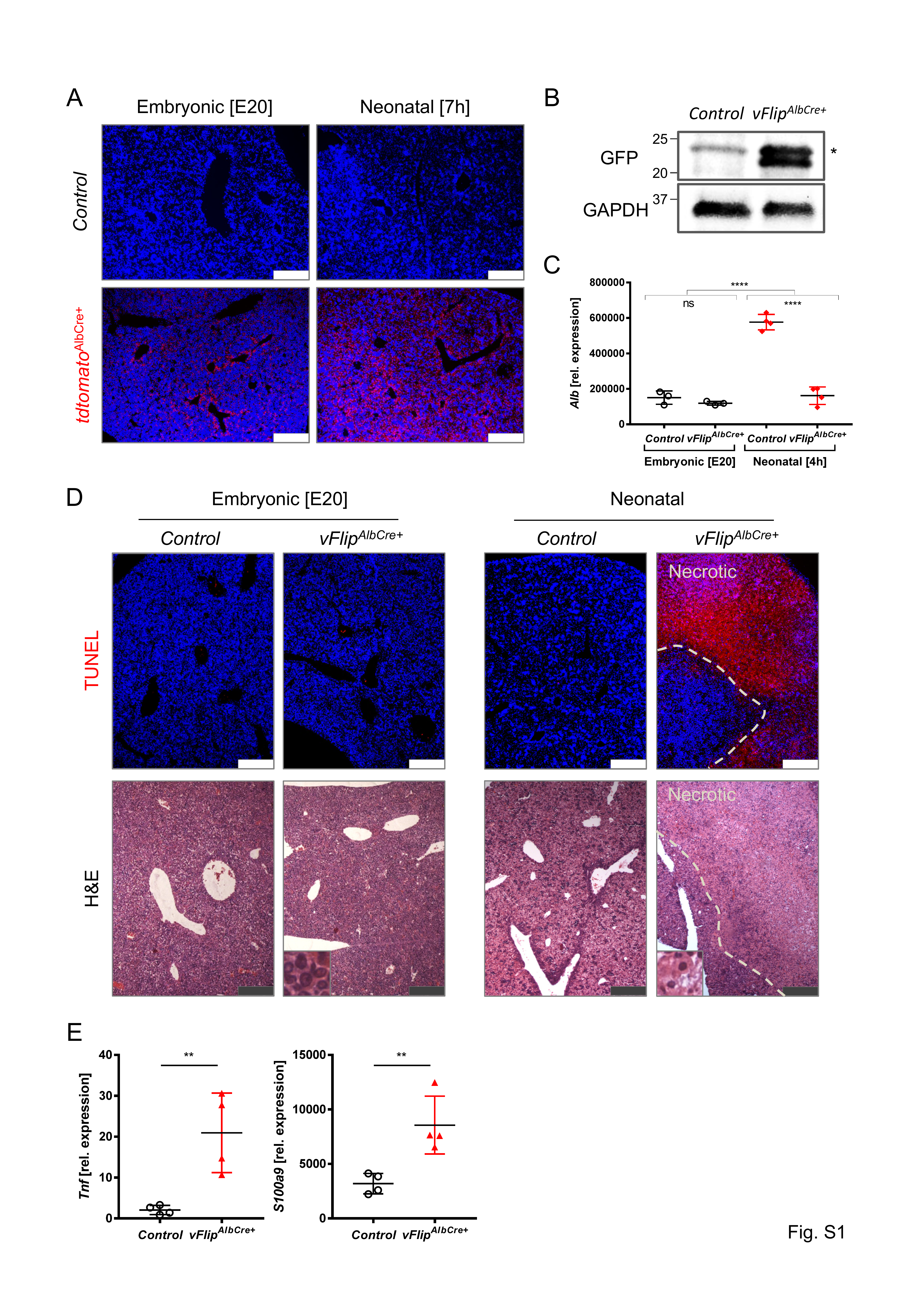

Supplement: Supplementary file 2 — Suppl. Fig. 1 [file 41419_2019_2115_MOESM2_ESM.png]

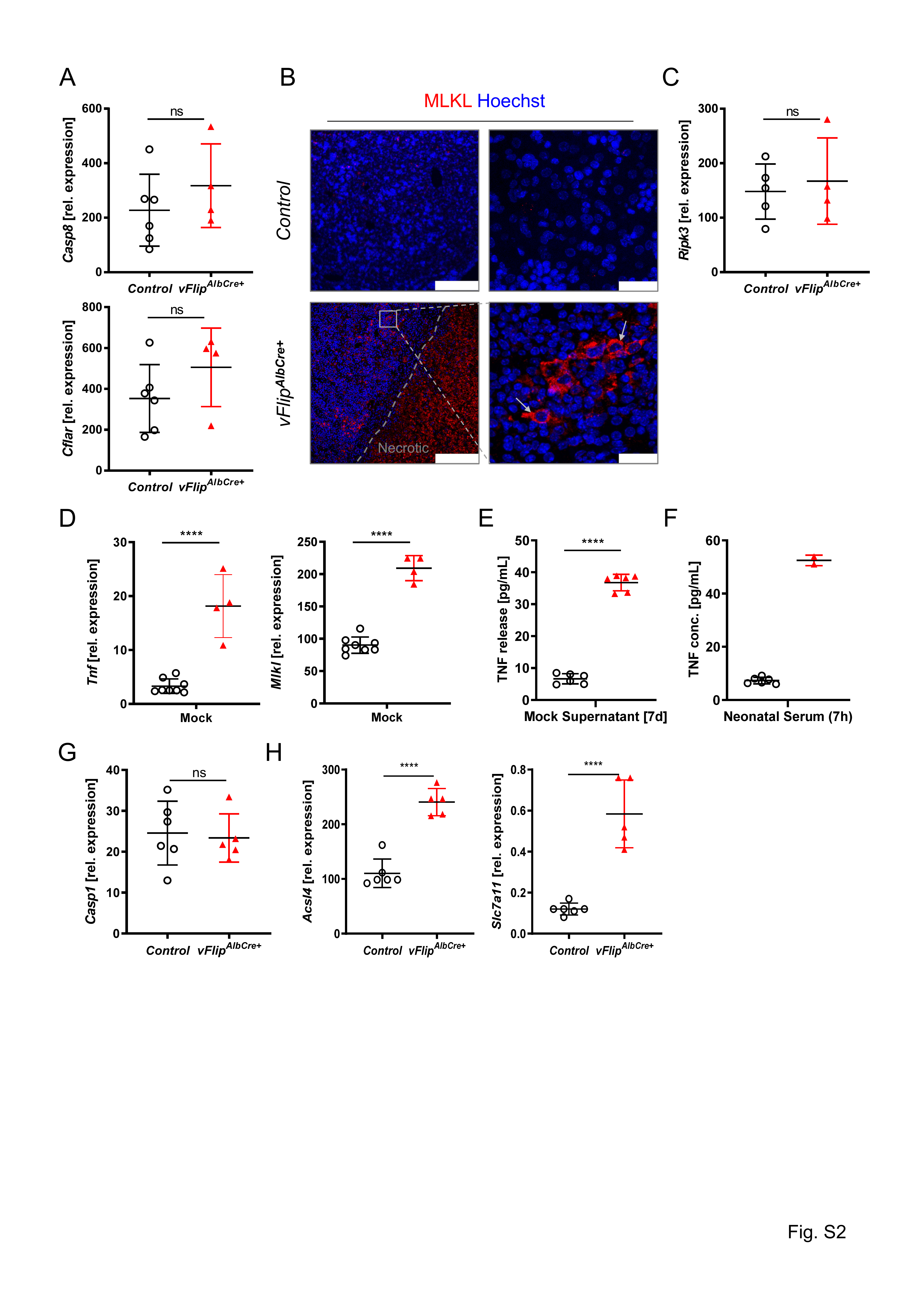

Supplement: Supplementary file 3 — Suppl. Fig. 2 [file 41419_2019_2115_MOESM3_ESM.png]

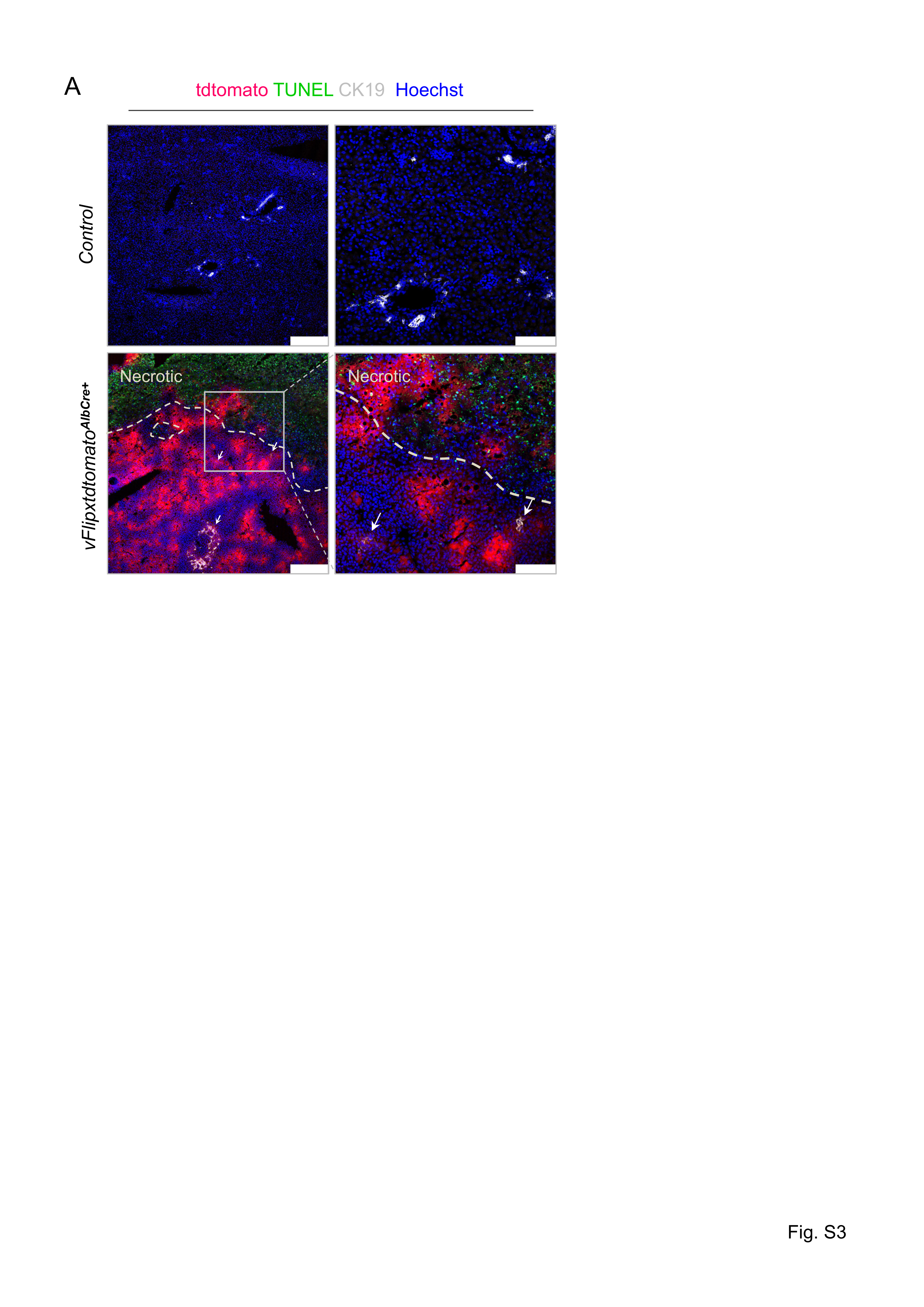

Supplement: Supplementary file 4 — Suppl. Fig. 3 [file 41419_2019_2115_MOESM4_ESM.png]

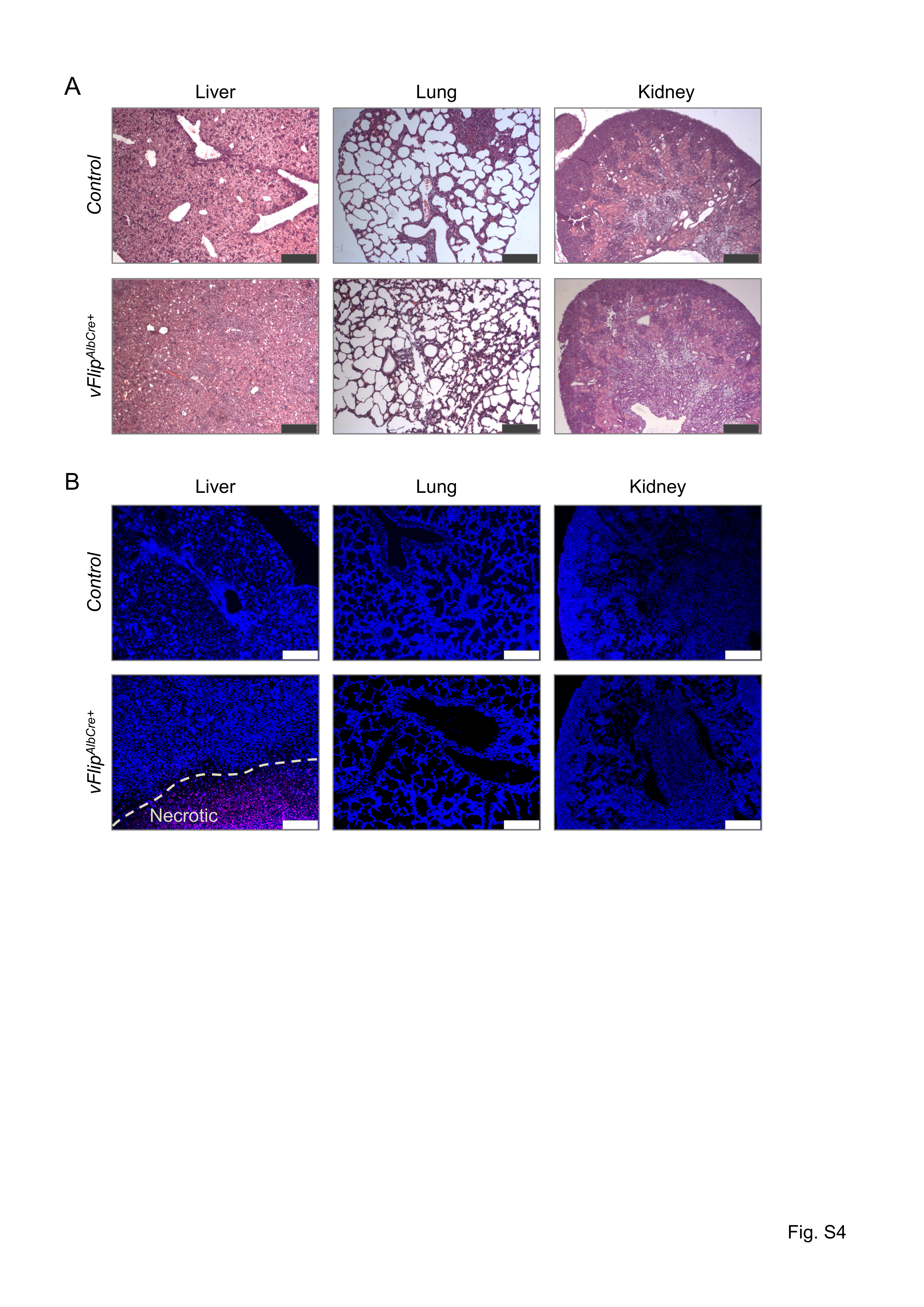

Supplement: Supplementary file 5 — Suppl. Fig. 4 [file 41419_2019_2115_MOESM5_ESM.png]
